# Muscarinic receptor signaling in the pathophysiology of asthma and COPD

**DOI:** 10.1186/1465-9921-7-73

**Published:** 2006-05-09

**Authors:** Reinoud Gosens, Johan Zaagsma, Herman Meurs, Andrew J Halayko

**Affiliations:** 1 Department of Molecular Pharmacology, University of Groningen, Groningen, The Netherlands; 2 Departments of Physiology & Internal Medicine, University of Manitoba, Winnipeg, MB, Canada; 3 Biology of Breathing Group, Manitoba Institute of Child Health, Winnipeg, MB, Canada

## Abstract

Anticholinergics are widely used for the treatment of COPD, and to a lesser extent for asthma. Primarily used as bronchodilators, they reverse the action of vagally derived acetylcholine on airway smooth muscle contraction. Recent novel studies suggest that the effects of anticholinergics likely extend far beyond inducing bronchodilation, as the novel anticholinergic drug tiotropium bromide can effectively inhibit accelerated decline of lung function in COPD patients. Vagal tone is increased in airway inflammation associated with asthma and COPD; this results from exaggerated acetylcholine release and enhanced expression of downstream signaling components in airway smooth muscle. Vagally derived acetylcholine also regulates mucus production in the airways. A number of recent research papers also indicate that acetylcholine, acting through muscarinic receptors, may in part regulate pathological changes associated with airway remodeling. Muscarinic receptor signalling regulates airway smooth muscle thickening and differentiation, both *in vitro *and *in vivo*. Furthermore, acetylcholine and its synthesizing enzyme, choline acetyl transferase (ChAT), are ubiquitously expressed throughout the airways. Most notably epithelial cells and inflammatory cells generate acetylcholine, and express functional muscarinic receptors. Interestingly, recent work indicates the expression and function of muscarinic receptors on neutrophils is increased in COPD. Considering the potential broad role for endogenous acetylcholine in airway biology, this review summarizes established and novel aspects of muscarinic receptor signaling in relation to the pathophysiology and treatment of asthma and COPD.

## Introduction

Acetylcholine is the primary parasympathetic neurotransmitter in the airways, and is traditionally associated with inducing airway smooth muscle contraction and mucus secretion. Parasympathetic activity is increased in airway inflammation, which is the basis for the use of anticholinergic therapy in asthma and chronic obstructive pulmonary disease (COPD) [1]. Anticholinergics constitute a particularly important bronchodilator therapy in COPD, as vagal tone appears to be the only reversible component of airflow limitation in this condition [1]. Recent evidence indicates that acetylcholine production in the airways is not restricted to the parasympathetic nervous system: acetylcholine is also released from non-neuronal origins such as the bronchial epithelium and inflammatory cells [2]. Furthermore, accumulating evidence suggests acetylcholine (either neuronal or non-neuronal) may play an essential regulatory role in the mechanisms that drive the structural changes in the airways, called airway remodeling, that are associated with chronic airway inflammation [3,4]. These recent findings indicate that acetylcholine, acting on muscarinic receptors, may contribute to the pathophysiology and pathogenesis of asthma and COPD to a much larger extent than is currently appreciated. This concept is underscored by findings that the recently introduced long-acting anticholinergic agent, tiotropium bromide [5], markedly inhibits accelerated lung function decline in COPD patients [6]. This article will review the established and novel muscarinic receptor signaling mechanisms in airway physiology, and discuss their involvement in the pathophysiology of asthma and COPD. Though nicotinic cholinergic receptors are present throughout the airways (see [7] for review), their function will not be discussed in view of the muscarinic receptor specificity of the current clinically used anticholinergics.

## Muscarinic receptor regulation of airway smooth muscle tone

Airway smooth muscle expresses abundant muscarinic M_2 _and M_3 _receptors, roughly in a 4:1 ratio [8]. Despite its lower expression levels, the G_q _coupled muscarinic M_3 _receptor is the primary subtype responsible for bronchial and tracheal smooth muscle contraction; this is evident from the functional affinities of a variety of subtype selective antagonists in airway tissues from diverse species, including humans [8-11] (Table 1). In addition, muscarinic M_3 _receptor -, but not M_2 _receptor-knockout mice lack both methacholine and vagally induced bronchoconstriction *in vivo *[12]. Nonetheless, some pharmacological studies have suggested a small role for G_i _– coupled M_2 _receptors in mediating airway smooth muscle contraction in the peripheral airways [13,14]. Muscarinic receptor regulation of airway smooth muscle tone is enhanced in asthma and COPD by two major mechanisms: first, increased expression and enhanced function of signaling molecules essential for muscarinic receptor mediated airway smooth muscle contraction; and second, exaggerated release of neuronal acetylcholine due to neuronal mechanisms associated with inflammation.

**Table 1:** Affinity profiles of selective and nonselective muscarinic receptor antagonists for muscarinic M_2 _and M_3 _receptors.

	M_2_*	M_3_#	
4-DAMP	7.8	9.0	[8, 11]
AQ-RA 741	8.3	7.5–6.6	[135]
Gallamine	6.5	4.3	[8]
DAU 5884	6.6	8.7	[14]
Methoctramine	7.5	6.5	[8]
Pirenzepine	6.2	6.8	[8, 11]
AF-DX 116	7.0	5.6–6.3	[8, 11]
Ipratropium	9.7^†^	9.7^†^	[5]

Tiotropium	10.7^†^	11.0^†^	[5]

* Data represent binding affinities (pK_i_) for cardiac muscarinic M_2 _receptors

# Data represent functional affinities (pA_2_) to methacholine-induced contraction of tracheal and bronchial preparations.

† Data represent binding affinities (pK_i_) to cloned human muscarinic receptor subtypes.

### Intracellular signaling in airway smooth muscle

**Figure 1:**
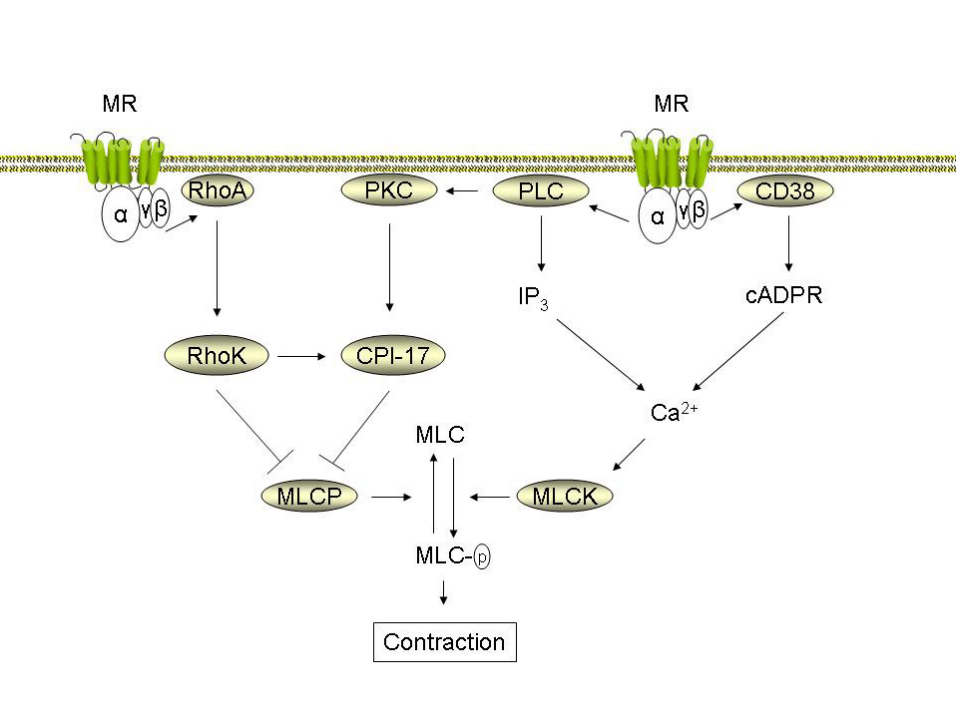
***Pathways central in muscarinic receptor mediated airway smooth muscle contraction***. Muscarinic receptor (MR) agonists induce contraction of airway smooth muscle by Ca^2+ ^dependent and Ca^2+ ^independent pathways. Through associated G_q _alpha subunits, the muscarinic M_3 _receptor activates phospholipase C (PLC), which releases inositol 1,4,5-trisphosphate (IP_3_) and diacylglycerol (DAG) after hydrolytic conversion of phosphatidylinositol-4,5-bisphosphate (PIP_2_). IP_3 _induces the release of Ca^2+ ^from internal sarcoplasmatic reticulum (SR) stores. Coupling of M_3 _receptor to CD38 through as yet undefined mechanisms contributes to the production of cyclic ADP ribose (cADPR) and the release of Ca^2+ ^through ryanodine receptor channels in the SR. Ca^2+ ^release increases free cytosolic Ca2+ and promotes calmodulin-dependent activation of myosin light chain kinase (MLCK). MLCK mediated phosphorylation of 20 kDa regulatory myosin light chain (MLC) in the contractile apparatus is an obligatory event to induce smooth muscle contraction. MLC phosphorylation level is also controlled by pathways that inhibit myosin light chain phosphatase (MLCP) and, thus enhance Ca^2+ ^sensitivity. PLC-derived DAG activates protein kinase C (PKC), leading to CPI-17 phosphorylation and downstream MLCP inhibition. Rho-kinase, which is activated by the monomeric G protein RhoA, both phosphorylates CPI-17 and inhibits MLCP directly. The expression and function of RhoA, CPI-17 and CD38 are increased by pro-inflammatory cytokines *in vitro *and in animal models of asthma and COPD *ex vivo *(see text).

Muscarinic receptors induce airway smooth muscle contraction through a number of intracellular signaling mechanisms; most of these are well described and have been reviewed extensively [4,15,16]. These include a number of recently identified cascades that are of specific interest to airway inflammation in asthma and COPD (Figure 1). Several researchers have postulated that enhanced Ca^2+ ^signaling underpins the genesis of obstructive airways diseases that are associated with airway hyperreactivity [17,18]. In part, this is based on observations from animal models, for example airway smooth muscle cells obtained from hyperresponsive Fisher rats show elevated Ca^2+ ^responses when compared to the less responsive Lewis rats [19]. Several studies have also shown that isolated airway smooth muscle preparations from asthma and COPD patients respond with increased maximal force generation to contractile stimulation *in vitro *[20-24]. Thus, intrinsic abnormalities that contribute to cholinergic hyperreactivity may exist in at least a proportion of asthmatics and COPD patients.

Although the altered expression of the postjunctional muscarinic M_3 _receptor on airway smooth muscle cells is not a feature of airway hyperreactivity to inhaled methacholine, changes in downstream signaling from these receptors may be a contributing factor [16]. In addition to activating phospholipase Cβ1 (PLC), which leads to inositol 1,4,5-trisphosphate (IP_3_) production necessary for triggering release of intracellular Ca^2+ ^stores, muscarinic receptors also regulate signaling pathways involving CD38, cyclic ADP ribose (cADPR) and ryanodine receptor channels that can play an important role in airway smooth muscle Ca^2+ ^homeostasis [25] (Figure 1). The CD38/cADPR pathway contributes significantly to muscarinic receptor mediated changes in lung compliance and resistance, as evident in CD38 knockout mice [26]. Initial studies suggest that this pathway may be activated selectively by muscarinic M_2 _receptors [27], though other studies suggest that muscarinic M_3_, rather than M_2 _receptors are coupled to cADPR production [28]. Several pro-inflammatory cytokines, including IL-1β [29], IL-13 [30], TNF-α [31] and IFN-γ [32] can increase CD38 expression, ADP-ribosyl cyclase activity, and Ca^2+ ^responses to cholinergic agonists in airway smooth muscle. TNF-α and IL-1β also increase G_i _and G_q _alpha protein expression in airway smooth muscle, which could account for increased Ca^2+ ^responses and contraction [33,34]. Furthermore, treatment of airway smooth muscle strips with IL-13 or TNF-α for extended periods, induces hyperresponsiveness to cholinergic agonists [35,36].

Contraction of airway smooth muscle is regulated by Ca^2+ ^dependent and Ca^2+ ^independent mechanisms. Ca^2+ ^independent mechanisms are characterized by augmented contraction at a fixed Ca^2+ ^concentration; this phenomenon is referred to as Ca^2+ ^sensitization [37]. Regulation of Ca^2+ ^sensitivity by cholinergic agonists is an important step in airway smooth muscle contraction (Figure 1). The RhoA/Rho-kinase cascade, a key regulatory pathway of Ca^2+ ^sensitivity in airway smooth muscle, can be activated by both muscarinic M_2 _and muscarinic M_3 _receptors [38,39]. RhoA and Rho-kinase augment agonist-induced contraction primarily by inactivating myosin light chain phosphatase (MLCP), although direct effects on myosin light chain phosphorylation and on actin cytoskeletal dynamics have also been described [40]. MLCP is inhibited by the direct phosphorylation of its regulatory myosin binding subunit by Rho kinase. MLCP is also inhibited by binding to the phosphoprotein CPI-17, which is targeted for phosphorylation by both Rho kinase and PKC (see [37] for detailed review on the role of Rho-kinase in airway hyperresponsiveness). The anti-spasmogenic effects of Rho-kinase inhibition are distinctly smaller than their relaxant effects on a pre-established cholinergic contraction, indicating that the RhoA/Rho-kinase pathway is particularly important in maintaining a sustained contraction to cholinergic agonists [41-43].

In experimental models of inflammatory airway disease, muscarinic receptor-linked signaling pathways that regulate Ca^2+ ^sensitivity of airway smooth muscle cells appear to be enhanced. Both RhoA and CPI-17 expression are increased in rats exposed to repeated allergen challenge [44,45]. Furthermore, allergic sensitization by itself, without subsequent allergen exposure, appears to be sufficient to induce an increase in RhoA expression [46]. Cytokines, including TNFα, have been identified as contributors to increased RhoA abundance [47]. In line with these observations, cholinergic agonist-induced RhoA translocation to the membrane, RhoA-mediated Ca^2+ ^sensitization, and contraction are increased in bronchial smooth muscle from rats and mice exposed to repeated allergen challenge [45,48,49]. Recent observations indicate that the same is true for cigarette smoke induced airway hyperresponsiveness in rat bronchial smooth muscle [50], which could be of significant importance to the pathophysiology of COPD. Effects of lipopolysaccharide (LPS) on cholinergic reactivity of airway smooth muscle have also been described [51]; however, it is yet to be established what mechanisms exactly mediate this change.

### Neuronal mechanisms

**Figure 2:**
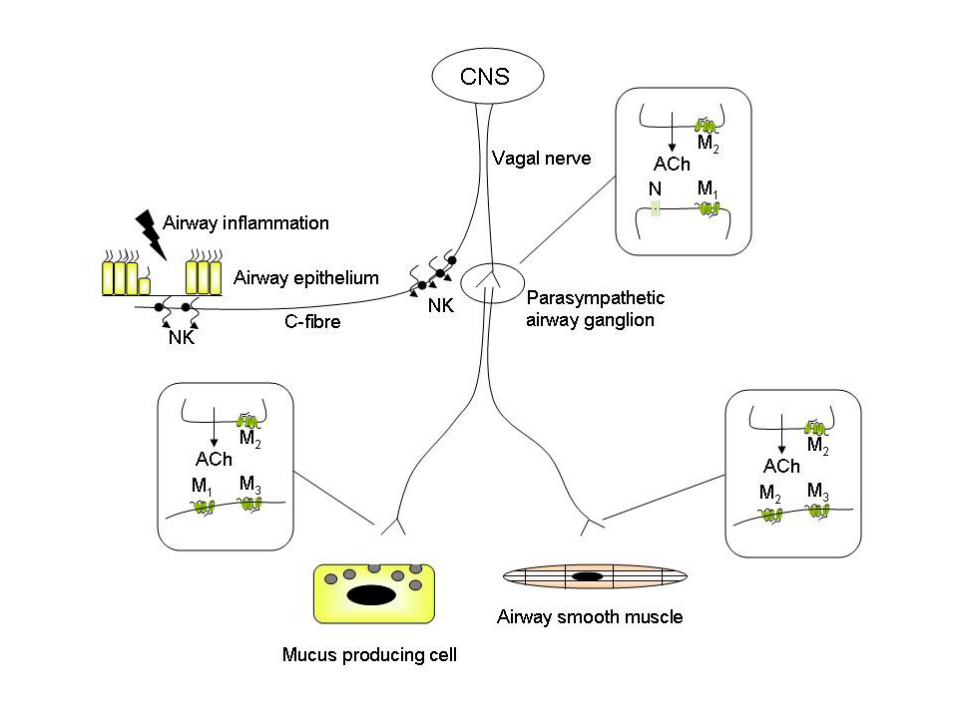
***Cholinergic receptors involved in neuronal acetylcholine release and function***. Neuronal acetylcholine release is regulated by a network of afferent and efferent airway nerves that interact with their surrounding cells. Afferent C-fibers project to the subepithelial region where they can be activated by inflammatory mediators and non-specific stimuli. In asthma, epithelial damage can expose sensory nerve endings to the airway lumen, potentiating their activation. Activated C-fibers secrete neurokinins (NK) that exert local effects and facilitate ganglionic neurotransmission (peripheral reflex arc). In addition, the activated C-fiber increases the output of the vagal nerve through regulation in the central nervous system (CNS) (central reflex arc). Neurotransmission in parasympathetic ganglia of the airway is mediated by acetylcholine through nicotinic (N) and muscarinic M_1 _receptors and can be markedly facilitated by inflammatory mediators (see text). Presynaptic muscarinic M_2 _autoreceptors inhibit acetylcholine release and are dysfunctional in airway inflammation. The postganglionic neurons project primarily to mucus producing cells and airway smooth muscle, where neurotransmission is regulated by muscarinic M_1_, M_2 _and M_3 _receptors, as indicated. As in the ganglia, prejunctional acetylcholine release is autoinhibited by muscarinic M_2 _receptors that are dysfunctional in airway inflammation. Acetylcholine release is augmented further by direct effects of inflammatory mediators on facilitatory presynaptic receptors. See the text for further detail.

In addition to postjunctional mechanisms that involve muscarinic receptor signaling in airway smooth muscle cells, neuronal mechanisms are important, and they also appear to be affected in inflammatory airways disease (Figure 2). Neuronal acetylcholine is synthesized by the enzyme choline acetyl transferase (ChAT), stored in vesicles, and released upon membrane depolarization. Once released, the functional effects of acetylcholine are terminated primarily by acetylcholinesterase (AChE) in the synaptic cleft. AChE activity is decreased in tracheal smooth muscle homogenates from ragweed pollen sensitized dogs [52]; this represents a mechanism to increase and prolong the action of acetylcholine on postjunctional target cells, such as airway smooth muscle cells, in allergic airways diseases. In addition, mediators of inflammation can enhance the release of acetylcholine from vagal nerve endings, an effect mediated through prejunctional facilitatory receptors. Examples include tachykinins, prostaglandins and thromboxane A_2 _[53]. Furthermore, the autoinhibitory prejunctional muscarinic M_2 _receptor, that limits acetylcholine release under normal conditions (Figure 2), is dysfunctional in several experimental models of airways disease including allergen exposure, viral infection, and ozone exposure [54,55]. M_2 _autoreceptors have also been reported to be dysfunctional in some, but not all asthmatics [56,57]. In addition, asthmatics with active viral infections show greater bronchodilator responses to inhaled anticholinergics, suggesting an increased vagal tone [58]. Nonetheless, in patients with stable COPD the M_2 _autoreceptor appears to function normally [59]. Distinct mechanisms underlie the M_2 _autoreceptor dysfunction. In guinea pigs, ozone and allergen-induced M_2 _dysfunction is mediated by eosinophils that are recruited to airway nerves, and secrete major basic protein, that acts as an allosteric muscarinic M_2 _receptor antagonist [55]. Viral infections, which may play a role in both asthma and COPD, induce M_2 _dysfunction through neuraminidases that cleave portions of the M_2 _receptor, and through as yet incompletely characterized mechanisms involving macrophages, CD8^+ ^lymphocytes, and possibly IFN-γ [60].

Cholinergic neurotransmission in the parasympathetic ganglia is regulated by nicotinic receptors in conjunction with muscarinic M_1 _receptors, whereas ganglionic release of acetylcholine from preganglionic nerves is under regulation of M_2 _autoreceptors [61,62] (Figure 2). Though the M_2 _autoreceptor can be dysfunctional in allergic airway inflammation, as described above, currently no evidence suggests that ganglionic muscarinic M_1 _receptor expression is altered [63]. However, several inflammatory mediators facilitate ganglionic neurotransmission, including tachykinins, histamine, bradykinin and prostaglandins [64]. Airway ganglia function to filter the signals from the rapidly firing preganglionic neurons; therefore faciliation of ganglionic transmission by inflammatory mediators is likely of significance in the regulation of airway tone [53].

Most of the afferent nerve fibres in the airways are C-fibers, which are present throughout the airways, from the larynx down to the lung parenchyma. C-fibers respond to stimuli such as heat and cold, but can also be activated by inflammatory mediators, resulting in reflex bronchoconstriction, mucus production and cough [65]. The localization of the reflex mechanism can be central and local, and may contribute considerably to the increased vagal tone in COPD, and to airway hyperreactivity in asthma and COPD [65,66]. Several inflammatory mediators, including histamine, prostanoids, thromboxane A_2_, bradykinin, serotonin and tachykinins are known to stimulate sensory nerve fibres [67]. Afferent sensory nerve endings project to the subepithelial layer in healthy airways, but may be exposed to the airway lumen upon the induction of epithelial damage by mediators such as eosinophil-derived major basic protein [68]. This is considered an important mechanism in the regulation of vagally mediated airway hyperrresponsiveness.

## Muscarinic receptor regulation of mucus hypersecretion

The production of airway mucus in the central airways is under cholinergic control, and plays an important role in asthma and COPD [69,70]. Airway mucus is a protective film that serves to prevent inhaled particles from damaging the airway epithelium. It is composed of electrolytes, water and contains high amounts of mucins [69]. Mucins are glycoproteins that are responsible for the high viscosity of mucus; the primary mucins found in airway mucus are of the MUC5AC and MUC5B isoforms. Mucus secreting cells in the central airways include goblet cells, which are embedded in the epithelium, and submucosal glands that are in connection to the airway lumen. Acetylcholine is the dominant neurotransmiter involved in mucus secretion in the central airways [71] (Figure 2). Thus, electrical field stimulation increases mucus production in bronchial preparations, which is sensitive to atropine and tetrodotoxin [72]. Airway submucosal glands are likely the primary source of this vagally regulated mucus production. Submucosal glands are innervated, and express functional muscarinic M_1 _and M_3 _receptors, roughly in a 1:2 ratio [73,74]. The muscarinic M_3 _receptor is the predominant receptor that mediates mucus secretion, whereas electrolyte and water secretion are probably mediated by muscarinic M_3 _receptors in cooperation with M_1 _receptors [72,75]. Goblet cells can also produce mucus in response to muscarinic receptor stimulation, albeit at relatively high concentrations of agonist [71].

Mucus hypersecretion is a pathological feature seen in both asthma and COPD that contributes significantly to airflow limitation by obstructing the airways [76]. The composition of mucus in asthma and COPD is generally altered, with higher expression of the low charge isoform MUC5B, particularly in COPD, and with the expression of small amounts of the insoluble MUC2; in COPD the ratio of mucus cells to serous cells in the submucosal glands is also increased [69]. Since mucus production in the central airways is, to a large extent, vagally mediated, acute airway inflammation can regulate mucus hypersecretion by augmenting acetylcholine release in the same way as described in the previous section. In addition, cholinergic receptor stimulation interacts synergistically with epidermal growth factor (EGF) on mucus cell activation in airway submucosal glands [77]. Since EGF is thought to regulate goblet cell hyperplasia and mucus gland hypertrophy in asthma and COPD [78,79], this may have additional implications for the effects of muscarinic receptors on this pathology. Indeed, muscarinic receptor stimulation transactivates the EGF receptor in conjunctival goblet cells, which is involved in mucin production by these cells [80]. In addition, based on observations in experimental animal models, repeated administration of the muscarinic agonists pilocarpine and methacholine can promote goblet cell hyperplasia and mucus gland hypertrophy [81]. This raises the real possibility that excessive release of endogenous acetylcholine could promote remodeling of mucus secreting cells in asthma and COPD, but this still needs to be assessed in human subjects.

## Muscarinic receptor regulation of airway inflammation

Traditionally, acetylcholine is not considered to regulate airway inflammation. Recruited inflammatory cells distribute throughout the lung, and are not primarily localized to vagal nerves. Early studies suggested that blood lymphocytes and peripheral polymorphonuclear leukocytes do not express functional muscarinic receptors [82]. This view is changing, however. Accumulating evidence demonstrates that acetylcholine and its synthesizing enzyme choline acetyltransferase (ChAT) are present not only in airway nerves, but localize to epithelial and endothelial cells, smooth muscle cells, lymphocytes, macrophages, mast cells, eosinophils and neutrophils as well [2,62]. Furthermore, rigorous investigation has now revealed that most inflammatory cells express functional muscarinic receptors (Table 2). These findings suggest that acetylcholine can regulate inflammatory processes by paracrine and/or autocrine mechanisms [83-86]. Notably, elevated levels of acetylcholine have been noted in skin biopsies from patients with atopic dermatitis, a condition often associated with bronchial asthma [87].

**Table 2:** Muscarinic regulation of (airway) inflammation.

**Cell type**	**Presence of muscarinic receptors, ChAT and/or acetylcholine**	**Functional effects of acetylcholine**	
T lymphocyte	Muscarinic receptors (M_1_-M_5 _*) ChAT Acetylcholine	Increased cytotoxicity Cytokine production Proliferation	[2, 83, 92]
B lymphocyte	Muscarinic receptors (M_1_-M_5 _*) ChAT Acetylcholine	Proliferation	[2, 83]
Mast cell	Muscarinic receptors (M_1_) ChAT Acetylcholine	Inhibition of histamine release	[2, 136, 137]
Neutrophil	Muscarinic receptors (M_1_/M_2_/M_3_) ChAT	Chemotaxis LTB_4 _production #	[93, 138]
Eosinophil	Muscarinic receptors (M_1_) ChAT	unknown	[93, 138]
Macrophage/monocyte	Muscarinic receptors (M_1_/M_2_/M_3_) ChAT Acetylcholine	LTB_4 _production #	[2, 83, 93]
Bronchial epithelium	Muscarinic receptors (M_1_/M_3_) ChAT Acetylcholine	Release of monocyte, eosinophil and neutrophil chemotactic factors	[95, 96, 98]
Airway smooth muscle	Muscarinic receptors (M_2_/M_3_) ChAT	Pro-inflammatory gene expression	[2, 8, 99]

* Further characterization is necessary: putative presence of muscarinic M_1 _– M_5 _receptors shows high variability and is based on their presence in mononuclear leukocytes [83].

# Presence of muscarinic receptors on macrophages and neutrophils suggests their involvement in LTB_4 _production by sputum cells [93].

Mild asthma and stable COPD show distinct patterns in the nature of airway inflammation. Stable COPD is characterized by pulmonary infiltration of neutrophils, cytotoxic (CD8^+^) T lymphocytes, monocytes and macrophages, whereas in mild asthma, Th2 (CD4^+^) lymphocytes and eosinophils show a distinct increase. The nature and extent of the airway inflammation are, however, dependent on severity of the disease, as in COPD and acute severe asthma both neutrophils and CD8^+ ^T lymphocytes are key contributors to disease pathology [88].

There is considerable evidence that the non-neuronal cholinergic system plays a role in lymphocytes, although its relative importance to airway physiology is not yet established. Mononuclear leukocytes, consisting mainly of lymphocytes, express ChAT mRNA and protein, contain ACh and express muscarinic and nicotinic receptors. The expression profile of muscarinic receptors shows high individual variability, although all 5 classes (M_1_-M_5_) of muscarinic receptors have been detected [83]. Muscarinic receptor agonists increase cytosolic Ca^2+ ^both in human T- and B-cell lines in an atropine-sensitive manner, and increase c-fos mRNA expression in response to the muscarinic agonist oxotremorine [89]. The latter effect is sensitive to 4-diphenylacetoxy-N-methylpiperidine (4-DAMP) methobromide, but not to pirenzepine or AF-DX 116, which is consistent with the involvement of muscarinic M_3 _receptors (Table 1). Furthermore, phytohemagglutinin (a T-cell activator) increased ChAT mRNA [90] and muscarinic M_5 _receptor expression [91] in stimulated mononuclear leukocytes. The significance of these observations is that muscarinic receptors and non-neuronal acetylcholine could contribute to lymphocyte proliferation and cytokine release, with obvious implications for airway inflammation in asthma and COPD. Furthermore, it is established that muscarinic receptors play an important role in regulating cytotoxicity of T lymphocytes [92]. Future studies are needed, however, to characterize the expression and function of the non-neuronal cholinergic system in lymphocytes that have infiltrated the lungs and in T lymphocytes that adopted a specific CD8^+ ^or CD4^+ ^phenotype

A recent study by Profita et al. [93] investigated the expression of muscarinic M_1_, M_2 _and M_3 _receptors in sputum cells obtained from healthy controls, smokers, and patients with COPD. In this study, all three subtypes of muscarinic receptors were observed in macrophages and neutrophils of all patient groups. M_1 _receptors were expressed in low abundance in eosinophils from COPD patients, but not from healthy controls. Importantly, the expression of muscarinic M_3 _receptors on macrophages is significantly increased in COPD patients, whereas muscarinic M_2 _receptor expression is decreased. The expression of muscarinic M_1 _receptors on macrophages, and the expression of M_1 _and M_3 _receptors on neutrophils tended to be increased, though this did not reach statistical significance. Functional studies showed that acetylcholine induced the release of significant amounts of leukotriene B_4 _and activated the p42/p44 MAP kinase pathway in sputum cells from COPD patients [93]. Neutrophil chemotactic activity induced by acetylcholine was also increased in COPD. These results are entirely consistent with a study demonstrating that bovine alveolar macrophages release eosinophil, monocyte and neutrophil chemotactic activities in response to acetylcholine, with probably a predominant involvement of leukotriene B_4 _[94]. These observations clearly reveal that regulated expression of muscarinic receptor subtypes is a feature of inflammatory cells that migrate to the airways, though the precise functional impact of dynamic receptor expression on these cells needs to be elucidated.

In addition to its direct effects on inflammatory cells, acetylcholine may also trigger chemokine and cytokine release from structural cells. Bronchial epithelial cells release eosinophil, monocyte and neutrophil chemotactic activity in response to acetylcholine [95,96]. Consistent with the previously mentioned findings, there appears to be an important role for leukotriene B_4 _in these effects. Acetylcholine is also known to induce the release of GM-CSF from human bronchial epithelial cells by a mechanism that involves nicotinic receptors [97]. Since the expression of non-neuronal acetylcholine is relatively high in bronchial epithelial cells [98], these results could implicate a role for epithelial acetylcholine in initiating inflammatory responses.

Muscarinic receptors on airway smooth muscle cells may play a profound role in regulating airway inflammation: a recent study demonstrates that the muscarinic receptor agonist carbachol increases inflammatory gene transcription in bovine tracheal smooth muscle strips [99]; quantitative RT-PCR analysis demonstrates that carbachol can modulate expression of a number of genes, including IL-8, cyclo-oxygenase (COX) 1 and 2 and urokinase type plasminogen activator (PLAU); and, carbachol markedly augments pro-inflammatory gene expression induced by sinusoidal length oscillation, with synergistic effects on IL-6, IL-8 and COX 2 and to a lesser extent PLAU and CCL-2 [99]. Collectively these studies suggest acetylcholine is an autocrine or paracrine hormone that may be involved in regulating inflammation at a number of cellular sites in the airways (Table 2). At this point, evidence is lacking however to indicate a direct involvement of non-neuronal acetylcholine in the pathophysiology of asthma and COPD and future studies are clearly warranted within this area.

## Muscarinic receptor regulation of airway remodeling

Chronic inflammatory conditions of the airways are usually associated with the development of structural changes of the airways; a phenomenon commonly referred to as airway remodeling. Airway remodeling is seen in both asthma and COPD, albeit the nature, localization and extent of the remodeling are variable (Table 3). Airway remodeling is progressive, both in asthma and COPD, and the extent of structural change correlates with disease severity [100-102]. Based on these considerations, it is believed that most structural changes, e.g. increased airways smooth muscle mass and mucus gland hypertrophy, contribute to a progressive increase in disease severity over time and to the irreversible decline in lung function in patients with chronic disease. Some structural changes on the other hand, including matrix deposition in the airway wall, are not necessarily detrimental, but may actually protect the diseased airway from airway closure by increasing airway wall stiffness [103,104]. Clearly a complex relationship exists between airway structure and function. Indeed, at present there is considerable ongoing research effort using in vitro, ex vivo, and in vivo systems to clarify key structural determinants of airway and lung function in health and disease.

**Table 3:** Airway remodeling in asthma and COPD. Muscarinic receptors and acetylcholine play significant roles in airway smooth muscle remodeling, and possibly in goblet cell hyperplasia and mucus gland hypertrophy. Their involvement in other aspects of airway remodeling is less well explored.

	**Asthma**		**COPD**	
Airway smooth	Hyperplasia	[121, 139]	Increased ASM mass	[140]
muscle (ASM)	Hypertrophy	[101, 139]	Hypercontractility	[23]
	Hypercontractility	[20-22, 24]		
Mucus production	Goblet cell hyperplasia Mucus gland hypertrophy	[141]	Goblet cell hyperplasia Mucus gland hypertrophy	[142]
Vasculature	Pulmonary vascular remodeling	[143]	Pulmonary vascular remodeling	[144-146]
Basement membrane	Thickened	[76]	Not thickened	[76]
Extracellular matrix	Subepithelial collagen deposition (Myo)fibroblast accumulation	[101, 147]	Airway wall fibrosis Loss of alveolar walls	[76]

Contractile agonists acting on G-protein coupled receptors are increasingly being recognized as key contributors to airway remodeling in asthma. Cysteinyl leukotrienes have received significant attention in this regard: the capacity for anti-leukotrienes to prevent allergen-induced airway inflammation, mucus production and occlusion, goblet cell hyperplasia, and most notably airway fibrosis and airway smooth muscle thickening have been described [105-109]. Acetylcholine, on the other hand, has not generally been considered to be a crucial determinant of structural changes in the airways. However, recent findings are changing this view. Indeed, there may be a prominent regulatory role for endogenous acetylcholine in promoting allergen-induced airway remodeling [4,110]. In the following sections the potential contribution of acetylcholine to specific components of airway remodelling are being discussed.

### Mesenchymal cell proliferation

Muscarinic receptor stimulation induces profound proliferation of primary cultured human lung fibroblasts [111]. In addition, though stimulation of muscarinic receptors is not sufficient to induce airway smooth muscle proliferation; muscarinic receptor agonists augment responses to epidermal growth factor (EGF) and platelet-derived growth factor (PDGF), in both human and bovine airway smooth muscle [112,113]. This augmentation is considerable: the dose-response curve to PDGF is shifted both upward and leftward, indicating that muscarinic receptors increase the mitogenic response to any PDGF concentration. Moreover, muscarinic agonists can potentiate the mitogenic response of myocytes in response to low concentrations of PDGF that would otherwise be insufficient to stimulate cell growth. The inhibitory profile of a range of subtype-selective antagonists (4-DAMP and DAU5884, but not gallamine) demonstrates the exclusive involvement of muscarinic M_3 _receptors in this effect [113] (Table 1). The importance of this potentiating effect is demonstrated *in vivo*: repeated exposures of sensitized guinea pigs to ovalbumin increases airway smooth muscle mass in the small, non-cartilaginous airways, which is largely inhibited by treatment with the anticholinergic agent tiotropium bromide [3]. In contrast, tiotropium bromide itself had no effect on airway smooth muscle mass, either in the cartilaginous or non-cartilaginous airways, which corroborates the *in vitro *findings that muscarinic receptor stimulation by itself is not sufficient to induce mitogenic responses.

**Figure 3:**
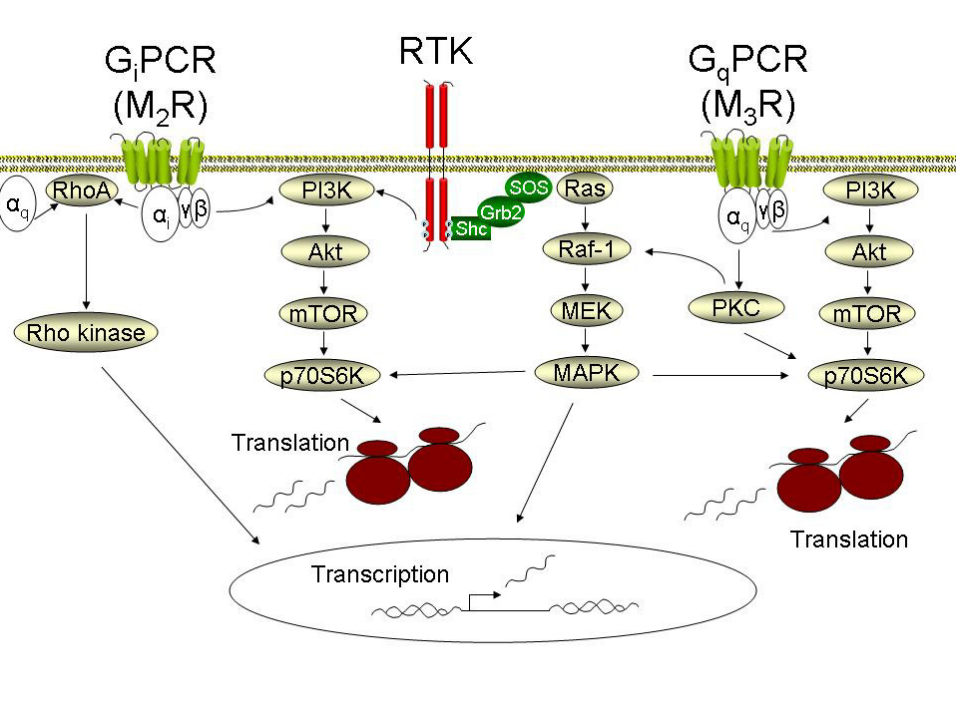
***Pathways involved in mesenchymal cell proliferation and differentiation induced by G protein coupled receptors (GPCRs)***. G protein coupled muscarinic receptors activate signaling cascades resulting in p42/p44 MAP kinase (MAPK), Rho-kinase and phosphatidyl-inositol-3-kinase (PI3K) activity. In addition, the signaling output of receptor tyrosine kinases (RTKs) is enhanced. Activation of the PI3K pathway appears to be particularly important in mesenchymal cell proliferation and differentiation. With Akt and mammalian target of rapamycin (mTOR) as signaling intermediates, PI3K activates p70S6K, which is involved in ribosome mediated protein translation. p42/p44 MAPK, activated by the sequential activation of Ras, Raf and MEK, also activates p70S6K and plays an important role in the induction of transcription factors involved in cell cycle progression. Rho-kinase activated transcription factors also play a central role in smooth muscle specific gene transcription, ultimately mediating the accumulation of contractile and contraction regulatory proteins. See the text for further detail.

The mechanisms that underlie the pro-mitogenic effects of muscarinic receptor stimulation have not yet been studied in detail. However, several intracellular signaling pathways have been identified that regulate the synergistic mitogenic interaction of other GPCR agonists with growth factors in airway smooth muscle (Figure 3). These pathways are not necessarily the same for every GPCR agonist; however, those studies do offer some important clues. The GPCR agonist thrombin augments EGF-induced proliferation through a pathway involving G_βγ_, phosphatidylinositol-3-kinase, Akt and p70S6kinase [114]. Although thrombin is mitogenic by itself and therefore presumably differs in its signaling profile from muscarinic receptor agonists, this pathway is of interest as synergistic activation of p70S6kinase by carbachol and EGF has been noted in human airway smooth muscle cells [112]. This mitogenic synergism might also involve PKC; this enzyme is responsible for the potentiating effects of the GPCR agonist bradykinin with EGF in airway smooth muscle [115], and regulates p70S6kinase activity [116,117]. PKC also regulates p42/p44 MAP kinase activation by muscarinic receptor agonists in airway smooth muscle [4]. The involvement of the small G protein RhoA in synergism induced by GPCR agonists and growth factors should also be considered [118], which is interesting given that RhoA expression is increased in animal models of asthma and COPD, as discussed before. Clearly, further studies are required to unravel in detail the signaling pathways involved in the potentiating effects of muscarinic receptor agonists on growth factor-induced airway smooth muscle proliferation.

### Mesenchymal cell differentiation

Mediators that drive contractile protein expression (e.g. TGF-β) are thought to play an important role in the differentiation of proliferating mesenchymal cells into mature airway smooth muscle cells, in airway smooth muscle cell hypertrophy, and in myofibroblast accumulation [119,120]. These responses, together with smooth muscle cell proliferation, contribute to increased airway smooth muscle mass in asthma, and possibly in COPD [101,119,121,122]. Increased expression of contractile apparatus associated proteins likely plays an important role in determining both airway bronchoconstrictor responsiveness and the extent of airway remodeling in asthma and COPD (Table 3) [110].

Mesenchymal cells from all individual airway wall compartments (adventitial fibroblasts, airway smooth muscle cells, mucosal fibroblasts) can be induced to acquire a more contractile phenotype, characterized by increases in smooth muscle specific protein expression, such as smooth muscle (sm)-α-actin, sm-myosin heavy chain (MHC) and desmin [123-125]. Induction of contractile protein gene transcription and protein translation in airway smooth muscle cells and fibroblasts is regulated by at least two pathways: the RhoA/Rho-kinase pathway and the phosphatidylinositol-3-kinase (PI3K)/mammalian target of rapamycin (mTOR)/p70S6kinase pathway [122,126] (Figure 3). There also appears to be a parallel and significant role for PKC as a modulator of the contribution of these pathways in the control of smooth muscle specific gene transcription, and protein accumulation [110,127]. Both the RhoA and PI3K pathways can be activated by muscarinic receptor agonists [43,112], and may be involved in mediating effects of muscarinic receptor agonists on SM22 and sm-MHC promoter activity [128]. Muscarinic receptor stimulation also leads to increased levels of sm-α-actin and sm-MHC mRNA in intact bovine tracheal smooth muscle strips, an effect that was also linked to mechanical strain applied to the strips [129]. Inhibition of PKC leads to an increase in RhoA-dependent transcription of SM22 and smMHC promoters [127]. Since PKC is strongly activated by muscarinic receptor agonists, therefore the contribution of this signaling pathway in the control of smooth muscle gene transcription needs to be established more clearly. In addition, Ca^2+ ^dependent pathways, induced by high concentrations of muscarinic receptor agonists, modulate smooth muscle specific contractile protein expression and contractility in organ-cultured bovine tracheal smooth muscle strips [130]. This indicates that the effects of muscarinic receptors are under tight control of multiple pathways. Future studies are clearly warranted in this area to better understand the interplay between the multiple pathways induced by muscarinic receptors, and their significance as determinants of airway smooth muscle differentiation and cellular hypertrophy.

Repeated exposures of sensitized guinea pigs to ovalbumin cause a ~4-fold increase in pulmonary sm-MHC expression with little effect on sm-α-actin expression [3]. Since sm-MHC is a far more stringent marker for mature airway smooth muscle cells than sm-α-actin, which is a more general marker for lung cells of mesenchymal origin [125], these results indicate that maturation of differentiated mesenchymal cells may have occurred in this model. Indeed, the contractile response of tracheal smooth muscle strips to methacholine was increased in the allergen challenged animals, whereas muscle mass in the large airways had not changed. Treatment of these animals with tiotropium bromide significantly inhibited the ovalbumin-induced sm-MHC expression and increases in tracheal contractility, indicating that endogenous acetylcholine contributes to these effects [3]. Collectively, these studies point to an important role for acetylcholine and muscarinic receptors in mesenchymal cell remodeling in allergic airways disease. The effects of muscarinic receptor antagonists on airway wall remodeling in animal models of COPD have not yet been investigated.

### Other aspects of airway remodeling

Evidence of the involvement of muscarinic receptor stimulation in other aspects of airway remodeling in asthma and COPD is scarce, mainly because this has not yet received sufficient attention to date. Since G-protein coupled receptor signaling has been associated with extracellular matrix production [131] and pulmonary vascular smooth muscle cell proliferation [132], effects of muscarinic receptor agonists on extracellular matrix remodeling and pulmonary vascular remodeling could be envisaged, though entirely speculative at this point. In addition, a role for muscarinic receptors in goblet cell hyperplasia and mucus gland hypertrophy has been postulated (see section on mucus hypersecretion). Future studies are clearly required to investigate the effects of muscarinic receptors on these aspects of airway remodeling.

## Therapeutic implications

Collectively, the observations we have discussed in this review suggest significant hitherto unexpected therapeutic implications. Thus, anticholinergic therapy could achieve far reaching and significant controller effects for chronic asthma and COPD that extend its capacity as reliever medications to promote bronchodilation. Based on the findings and considerations presented above, it could be envisaged that anticholinergics inhibit airway inflammation and limit airway remodeling, retarding the progressive decline in lung function in asthma and COPD patients.

Though previous studies using ipratropium bromide indicate no improvement in the annual decline in lung function in patients with obstructive airways diseases [132], these therapeutic outcomes may relate to limitations of this drug. Ipratropium bromide is short-acting, whereas the recently introduced anticholinergic agent tiotropium bromide is long-acting and more potent. In addition, tiotropium bromide has a considerably longer relative half-life of dissociation from muscarinic M_3 _and M_1 _receptors than from muscarinic M_2 _receptors, making the drug 'kinetically selective' for M_3 _and M_1 _receptors [5,133,134]. It is still debatable as to whether this kinetic selectivity is clinically important; however, since M_2 _autoreceptor blockade is associated with enhanced acetylcholine outflow from the vagal nerve, whereas muscarinic M_3 _receptor blockade inhibits most of the postjunctional effects of acetylcholine (as described above) some beneficial effects of this property could be envisaged.

Indeed, a recent study indicates that tiotropium bromide induces a marked reduction in lung function decline of COPD patients [6]. Although this study was retrospective, the results of this study were remarkable: the mean decline in FEV_1 _in one year was 58 ml in the placebo group vs. 12 ml in the tiotropium group. This reduction has not been observed with ipratropium bromide in COPD patients [133]. Tiotropium bromide is also superior to ipratropium bromide on other aspects, both with respect to spirometry, health related quality of life and number of exacerbations in COPD patients [134]. In view of our own recent findings using a guinea pig model of ongoing allergic asthma [3], showing that tiotropium bromide protects against allergen-induced increases in airway smooth muscle thickening, contractile protein accumulation and tracheal hypercontractility, the drug tiotropium bromide might also be effective in slowing or preventing airway remodeling in chronic asthma. Future studies are required to translate these findings to asthma patients, however.

## Conclusion

Acetylcholine is a parasympathetic neurotransmitter and an autocrine or paracrine hormone that regulates airway smooth muscle contraction, mucus production, airway inflammation and airway remodeling. The release of acetylcholine, and the expression of several effector systems central in muscarinic regulation of airway function are enhanced in asthma and COPD, suggesting that the effects of acetylcholine could contribute significantly to the pathophysiology of these obstructive airways diseases. Recent clinical and experimental findings support this hypothesis, suggesting that anticholinergics, most notably the long-acting tiotropium bromide, could achieve reductions in airway remodeling and lung function decline in addition to its effects as a bronchodilator.

## Authors' contributions

RG participated in the design of the article and drafted the manuscript. AJH, JZ and HM participated in the design of the article, assisted in drafting the manuscript and revised it critically for important intellectual content. All authors approved the final manuscript.
